# Supplementary material for: Effect of paternal-maternal parenting styles on college students’ internet addiction of different genders: The mediating role of life satisfaction
Source: PLoS One. 2024 May 14;19(5):e0303554. doi: 10.1371/journal.pone.0303554 (PMC11093304; doi:10.1371/journal.pone.0303554)
Supplement: S1 File — (DOCX) [file pone.0303554.s001.docx]

| Variable | Item | Mean | Standard Deviation | Varience |
| --- | --- | --- | --- | --- |
| internet addiction | IA | 58.5676 | 11.59720 | 134.495 |
|  | SymC | 10.9092 | 2.55419 | 6.524 |
|  | SymW | 11.6708 | 2.52491 | 6.375 |
|  | SymT | 9.6339 | 2.07719 | 4.315 |
|  | RPIH | 15.3246 | 3.58029 | 12.818 |
|  | RPTM | 11.0291 | 2.76653 | 7.654 |
| Item | IA1 | 2.5781 | 0.75446 | 0.569 |
|  | IA2 | 2.6209 | 0.71177 | 0.507 |
|  | IA3 | 2.6095 | 0.70924 | 0.503 |
|  | IA4 | 2.4685 | 0.72109 | 0.520 |
|  | IA5 | 2.1975 | 0.68952 | 0.475 |
|  | IA6 | 2.6536 | 0.70266 | 0.494 |
|  | IA7 | 1.9545 | 0.70498 | 0.497 |
|  | IA8 | 2.0062 | 0.85486 | 0.731 |
|  | IA9 | 2.3233 | 0.73324 | 0.538 |
|  | IA10 | 2.0289 | 0.65866 | 0.434 |
|  | IA11 | 2.0021 | 0.67796 | 0.460 |
|  | IA12 | 2.0949 | 0.69821 | 0.488 |
|  | IA13 | 2.2727 | 0.74878 | 0.561 |
|  | IA14 | 2.1095 | 0.74364 | 0.553 |
|  | IA15 | 2.2433 | 0.73895 | 0.546 |
|  | IA16 | 2.3550 | 0.74695 | 0.558 |
|  | IA17 | 2.1445 | 0.72466 | 0.525 |
|  | IA18 | 2.3488 | 0.72460 | 0.525 |
|  | IA19 | 2.3998 | 0.72788 | 0.530 |
|  | IA20 | 2.0900 | 0.69542 | 0.484 |
|  | IA21 | 2.2658 | 0.71562 | 0.512 |
|  | IA22 | 2.3079 | 0.71788 | 0.515 |
|  | IA23 | 2.0526 | 0.72377 | 0.524 |
|  | IA24 | 2.0475 | 0.70028 | 0.490 |
|  | IA25 | 2.0661 | 0.76657 | 0.588 |
|  | IA26 | 2.3261 | 0.78024 | 0.609 |

| Variable | Item | Mean | Standard Deviation | Varience |
| --- | --- | --- | --- | --- |
| Every dimension of father’s parenting styles | MotEW | 52.3058 | 11.38205 | 129.551 |
|  | MotIP | 31.7156 | 6.68297 | 44.662 |
|  | MotR | 12.5703 | 3.97182 | 15.775 |
|  | MotP | 12.9689 | 4.36185 | 19.026 |
|  | MotPr | 11.0396 | 3.00008 | 9.000 |
| Item | MPS1 | 1.9043 | 0.68494 | 0.469 |
|  | MPS2 | 3.0208 | 0.84857 | 0.720 |
|  | MPS3 | 2.5954 | 1.05825 | 1.120 |
|  | MPS4 | 3.1259 | 0.87746 | 0.770 |
|  | MPS5 | 1.7430 | 0.74456 | 0.554 |
|  | MPS6 | 2.6212 | 0.88939 | 0.791 |
|  | MPS7 | 2.8067 | 0.90206 | 0.814 |
|  | MPS8 | 2.1377 | 0.98485 | 0.970 |
|  | MPS9 | 2.9031 | 0.90088 | 0.812 |
|  | MPS10 | 2.0521 | 0.79816 | 0.637 |
|  | MPS11 | 1.7823 | 0.84485 | 0.714 |
|  | MPS12 | 2.0894 | 0.87779 | 0.771 |
|  | MPS13 | 1.8264 | 0.78680 | 0.619 |
|  | MPS14 | 1.8845 | 0.79737 | 0.636 |
|  | MPS15 | 2.8384 | 0.93911 | 0.882 |
|  | MPS16 | 2.3375 | 0.89009 | 0.792 |
|  | MPS17 | 1.4356 | 0.68442 | 0.468 |
|  | MPS18 | 1.9012 | 0.79048 | 0.625 |
|  | MPS19 | 1.8399 | 0.86534 | 0.749 |
|  | MPS20 | 3.5343 | 0.72043 | 0.519 |
|  | MPS21 | 1.9023 | 0.81320 | 0.661 |
|  | MPS22 | 2.0252 | 1.00382 | 1.008 |
|  | MPS23 | 1.5437 | 0.66615 | 0.444 |
|  | MPS24 | 2.3757 | 0.86416 | 0.747 |
|  | MPS25 | 2.8150 | 0.89342 | 0.798 |
|  | MPS26 | 1.3763 | 0.65131 | 0.424 |
|  | MPS27 | 1.5094 | 0.72118 | 0.520 |
|  | MPS28 | 1.4568 | 0.68135 | 0.464 |
|  | MPS29 | 2.6833 | 0.87390 | 0.764 |
|  | MPS30 | 2.6954 | 0.93459 | 0.873 |
|  | MPS31 | 2.8441 | 0.86130 | 0.742 |
|  | MPS32 | 2.9074 | 0.85172 | 0.725 |
|  | MPS33 | 3.0218 | 0.88145 | 0.777 |
|  | MPS34 | 1.4125 | 0.66769 | 0.446 |
|  | MPS35 | 1.5573 | 0.75239 | 0.566 |
|  | MPS36 | 1.6333 | 0.75657 | 0.572 |
|  | MPS37 | 2.1771 | 0.93798 | 0.880 |
|  | MPS38 | 1.7028 | 0.81669 | 0.667 |
|  | MPS39 | 1.6893 | 0.75415 | 0.569 |
|  | MPS40 | 2.1594 | 0.86370 | 0.746 |
|  | MPS41 | 2.1521 | 0.86680 | 0.751 |
|  | MPS42 | 2.6490 | 0.96668 | 0.934 |
|  | MPS43 | 1.2682 | 0.54107 | 0.293 |
|  | MPS44 | 2.6736 | 0.80726 | 0.652 |
|  | MPS45 | 1.8044 | 0.79284 | 0.629 |
|  | MPS46 | 2.1021 | 0.87760 | 0.770 |
|  | MPS47 | 1.5846 | 0.74117 | 0.549 |
|  | MPS48 | 2.1491 | 0.81248 | 0.660 |
|  | MPS49 | 1.4172 | 0.73928 | 0.547 |
|  | MPS50 | 2.2258 | 0.92134 | 0.849 |
|  | MPS51 | 1.3650 | 0.66583 | 0.443 |
|  | MPS52 | 1.4259 | 0.67661 | 0.458 |
|  | MPS53 | 1.3073 | 0.62913 | 0.396 |
|  | MPS54 | 1.8314 | 0.76862 | 0.591 |
|  | MPS55 | 1.4583 | 0.67974 | 0.462 |
|  | MPS56 | 2.6181 | 0.85055 | 0.723 |
|  | MPS57 | 1.8073 | 0.83613 | 0.699 |
|  | MPS58 | 1.4083 | 0.65933 | 0.435 |
|  | MPS59 | 1.8707 | 0.77884 | 0.607 |
|  | MPS60 | 2.8925 | 0.93024 | 0.865 |
|  | MPS61 | 2.6847 | 0.85699 | 0.734 |
|  | MPS62 | 1.4740 | 0.71174 | 0.507 |
|  | MPS63 | 3.1145 | 0.88313 | 0.780 |
|  | MPS64 | 2.0759 | 0.99924 | 0.998 |
|  | MPS65 | 1.3232 | 0.63196 | 0.399 |
|  | MPS66 | 1.9885 | 0.89331 | 0.798 |

| Variable | Item | Mean | Standard Deviation | Variance |
| --- | --- | --- | --- | --- |
| Every dimension of father’s parenting styles | FatEW | 51.6302 | 11.30153 | 127.725 |
|  | FatOI | 18.6959 | 4.11910 | 16.967 |
|  | FatP | 17.6610 | 5.68456 | 32.314 |
|  | FatPr | 10.0182 | 3.44696 | 11.882 |
|  | FatR | 9.2443 | 2.93327 | 8.604 |
|  | FatOP | 9.8350 | 2.47382 | 6.120 |
| Item | FPS1 | 1.7242 | 0.61547 | 0.379 |
|  | FPS2 | 2.9044 | 0.88301 | 0.780 |
|  | FPS3 | 2.5906 | 1.05879 | 1.121 |
|  | FPS4 | 3.0969 | 0.87287 | 0.762 |
|  | FPS5 | 1.6774 | 0.73651 | 0.542 |
|  | FPS6 | 2.5753 | 0.89818 | 0.807 |
|  | FPS7 | 2.8224 | 0.90240 | 0.814 |
|  | FPS8 | 2.1122 | 1.00052 | 1.001 |
|  | FPS9 | 2.8895 | 0.89875 | 0.808 |
|  | FPS10 | 2.0280 | 0.79089 | 0.626 |
|  | FPS11 | 1.5806 | 0.76816 | 0.590 |
|  | FPS12 | 2.0010 | 0.85186 | 0.726 |
|  | FPS13 | 1.7451 | 0.77389 | 0.599 |
|  | FPS14 | 1.7549 | 0.76286 | 0.582 |
|  | FPS15 | 2.7266 | 0.95613 | 0.914 |
|  | FPS16 | 2.2422 | 0.85403 | 0.729 |
|  | FPS17 | 1.4563 | 0.70540 | 0.498 |
|  | FPS18 | 1.8359 | 0.78947 | 0.623 |
|  | FPS19 | 1.6819 | 0.79508 | 0.632 |
|  | FPS20 | 3.4231 | 0.78864 | 0.622 |
|  | FPS21 | 1.7121 | 0.75266 | 0.567 |
|  | FPS22 | 1.9602 | 1.00025 | 1.001 |
|  | FPS23 | 1.5042 | 0.65819 | 0.433 |
|  | FPS24 | 2.3212 | 0.86438 | 0.747 |
|  | FPS25 | 2.7692 | 0.89093 | 0.794 |
|  | FPS26 | 1.3614 | 0.63559 | 0.404 |
|  | FPS27 | 1.4304 | 0.66238 | 0.439 |
|  | FPS28 | 1.4091 | 0.64734 | 0.419 |
|  | FPS29 | 2.6258 | 0.89007 | 0.792 |
|  | FPS30 | 2.5597 | 0.97022 | 0.941 |
|  | FPS31 | 2.8162 | 0.87077 | 0.758 |
|  | FPS32 | 2.8847 | 0.85138 | 0.725 |
|  | FPS33 | 2.9324 | 0.90467 | 0.818 |
|  | FPS34 | 1.4183 | 0.67477 | 0.455 |
|  | FPS35 | 1.4636 | 0.70873 | 0.502 |
|  | FPS36 | 1.5563 | 0.71581 | 0.512 |
|  | FPS37 | 2.1374 | 0.92014 | 0.847 |
|  | FPS38 | 1.6119 | 0.76696 | 0.588 |
|  | FPS39 | 1.6621 | 0.74387 | 0.553 |
|  | FPS40 | 2.1250 | 0.84821 | 0.719 |
|  | FPS41 | 2.1217 | 0.87332 | 0.763 |
|  | FPS42 | 2.5401 | 0.96882 | 0.939 |
|  | FPS43 | 1.1983 | 0.44943 | 0.202 |
|  | FPS44 | 2.6345 | 0.81734 | 0.668 |
|  | FPS45 | 1.7370 | 0.78578 | 0.617 |
|  | FPS46 | 2.0000 | 0.84882 | 0.720 |
|  | FPS47 | 1.5156 | 0.69883 | 0.488 |
|  | FPS48 | 2.0229 | 0.79432 | 0.631 |
|  | FPS49 | 1.3766 | 0.66752 | 0.446 |
|  | FPS50 | 2.2048 | 0.90585 | 0.821 |
|  | FPS51 | 1.3663 | 0.64755 | 0.419 |
|  | FPS52 | 1.4158 | 0.68228 | 0.466 |
|  | FPS53 | 1.3107 | 0.64941 | 0.422 |
|  | FPS54 | 1.7869 | 0.76499 | 0.585 |
|  | FPS55 | 1.4428 | 0.69407 | 0.482 |
|  | FPS56 | 2.6040 | 0.87293 | 0.762 |
|  | FPS57 | 1.7898 | 0.85219 | 0.726 |
|  | FPS58 | 1.3815 | 0.65859 | 0.434 |
|  | FPS59 | 1.8046 | 0.77303 | 0.598 |
|  | FPS60 | 2.8033 | 0.94125 | 0.886 |
|  | FPS61 | 2.6944 | 0.86701 | 0.752 |
|  | FPS62 | 1.4542 | 0.71510 | 0.511 |
|  | FPS63 | 3.0395 | 0.91824 | 0.843 |
|  | FPS64 | 2.0431 | 1.00683 | 1.014 |
|  | FPS65 | 1.3122 | 0.61349 | 0.376 |
|  | FPS66 | 1.7944 | 0.82938 | 0.688 |

| Variable | Item | Mean | Standard Deviation | Variance |
| --- | --- | --- | --- | --- |
| life satisfaction | LS | 20.2454 | 6.30627 | 39.769 |
| Item | LS1 | 4.2052 | 1.49373 | 2.231 |
|  | LS2 | 4.0828 | 1.46646 | 2.150 |
|  | LS3 | 4.2446 | 1.43755 | 2.067 |
|  | LS4 | 4.3261 | 1.53641 | 2.361 |
|  | LS5 | 3.3868 | 1.56282 | 2.442 |
