# Supplementary material for: Effect of paternal-maternal parenting styles on college students’ internet addiction of different genders: The mediating role of life satisfaction
Source: PLoS One. 2024 May 14;19(5):e0303554. doi: 10.1371/journal.pone.0303554 (PMC11093304; doi:10.1371/journal.pone.0303554)
Supplement: Attachment — Submitted filename: review comments.docx [file pone.0303554.s003.docx]

**Review Comments**

Thank you very much for the opportunity to participate in reviewing your manuscript, which is an interesting topic. But I still have some concerns that need to be addressed, otherwise the manuscript will not be published in PLOS ONE:

**Abstract:**

1. Please unify the key terms. Is it Internet use, Internet use behavior or Internet addiction? These are several different concepts and terms. Please check the abstract and the full manuscript carefully. Please use one special term uniformly. This is a very serious matter.
2. Lines 21 – 22 of the abstract, is there a grammatical mistake in this sentence? I don't understand.

**Introduction：**

3. The quotation of the whole introduction part is not in line with the academic norms, and the non-personal views should be supported by the literature.
4. Lines 41-42, as far as I know, there is not a few literature related to parenting style, life satisfaction and Internet addiction. Please cite the appropriate literature of recent years to support this statement.
5. The logic of the Introduction needs to be adjusted and supplemented, and several subheadings need to be clarified to clearly state the relationships between the various variables, the theoretical foundations, and the hypotheses of the study.
6. Lines 97 and 99, “Previous studies........ , multiple studies......”, but to cite only one study as support is very loose. This kind of situation occurs in many places in the manuscript, and this problem is very bad and needs to be paid attention to by the author.
7. Please clarify the research questions and the study hypotheses.
8. If the hypothesis model is to explore the dimensions, the literature review on the relationship between the dimensions and the main variables needs to be supplemented in the Introduction.

**Methods**

9. The data for this study were collected from September to October 2021, and it is currently 2024, so the timeliness of the data is worth considering. In addition, what is the serial number of ethical approval?
10. The data is collected through the psychological system. Is there a valid link to this system?
11. Please provide sample items for each scale.
12. Please specify the process of questionnaire collection and how to identify invalid questionnaires?
13. Authors only provide reliability, whether the validity meets the criteria, please add.

**Results**

14. Please provide valid references for the standard values of all indicators in the Results section, such as Common method deviation test (180 lines), etc.

**Others:**

15. Please add practical suggestions.
16. Please specify exactly how future research should proceed in the Limitations.
17. The findings, contributions and implications of this study need to be summarized in the concluding section.

In short, there are many errors in the translation of the manuscript that need to be improved, no DOI number in many references (for example, [14], [21]), confusion of proper nouns, the manuscript needs to be carefully revised, and the current quality does not meet the academic level of PLOS ONE journals.
