# Supplementary material for: Effect of paternal-maternal parenting styles on college students’ internet addiction of different genders: The mediating role of life satisfaction
Source: PLoS One. 2024 May 14;19(5):e0303554. doi: 10.1371/journal.pone.0303554 (PMC11093304; doi:10.1371/journal.pone.0303554)
Supplement: Attachment — Submitted filename: Response to Reviewers.docx [file pone.0303554.s004.docx]

To Editor,

We sincerely thank the editor and all reviewers for valuable feedback that we have used to improve the quality of our manuscript. As you are concerned, there are several problems that need to be addressed. According to your nice suggestions, we have made extensive corrections to our previous manuscript, the detailed corrections are listed below. The reviewer comments are laid out below in **bold font** and specific concerns have been numbered. And changes/additions to the manuscript are identified using the “Track Changes” option in Microsoft Word.

**#Reviewer 1:**

**Comments 1: Please unify the key terms. Is it Internet use, Internet use behavior or Internet addiction? These are several different concepts and terms. Please check the abstract and the full manuscript carefully. Please use one special term uniformly. This is a very serious matter.**

**Response to comments 1:**

Thank you for pointing out the omissions in the manuscript. We have carefully revised and unify the concept. In this study, the main research variable was internet addition, therefore, we have carefully revised the manuscript regarding word usage errors.

Revised content:

See lines: 14, 18, 23, 27, 29, 66, 123, 166, 198, 201, 207, 211, 214, 220, 221, 223, 465, 496, 501-503, 510, 605, 607. (revised manuscript with track changes)

See lines:14, 18, 23, 25, 27, 53, 100, 129, 150, 153, 158, 161, 164, 169, 171, 173, 378, 408, 412-414, 421, 506, 507. (manuscript)

**Comments 2: Lines 21 – 22 of the abstract, is there a grammatical mistake in this sentence? I don't understand.**

**Response to comments 2:**

We would like to thank the reviewer for pointing out this issue. The error in lines 21-22 has been modified. We have changed this sentence to: For male college students, life satisfaction mediated the two dimensions of parenting styles (the father’s emotional warmth, the father’s overprotection) and internet addiction.

Revised content:

See lines:20-23. (revised manuscript with track changes)

See lines:20-22. (manuscript)

**Comments 3: The quotation of the whole introduction part is not in line with the academic norms, and the non-personal views should be supported by the literature.**

**Response to comments 3:**

We sincerely appreciate the valuable comments. We have checked the literature carefully. Moreover, we have added more references into **Introduction** to support this idea.

References:

2.Young KS. Internet addiction: The emergence of a new clinical disorder. CyberPsychol Behav 1998; 1(3), 237–244. <https://doi.org/10.1089/cpb.1998.1.237>

5.Li L, Xu DD, Chai JX, Wang D, Li L, Zhang L, et.al. Prevalence of internet addiction disorder in Chinese university students: A comprehensive meta-analysis of observational studies. J Behav Addict. 2018;7(3):610-623. <https://doi.org/10.1556/2006.7.2018.53>. PMID: 30010411.

6.Duc TQ, Chi VTQ, Huyen NTH, Quang PN, Thuy BT, Nguyen Di K. Growing propensity of internet addiction among Asian college students: meta-analysis of pooled prevalence from 39 studies with over 50,000 participants. Public Health. 2024; 227: 250-258. <https://doi.org/10.1016/j.puhe.2023.11.040>. PMID: 38262230.

7.Zhao Y, Zhang K, Griffiths MD. Serial mediation roles of Alexithymia and loneliness in the association between family function and internet addiction among Chinese college students. Front Psychol. 2022; 13: 874031. <https://doi.org/10.3389/fpsyg.2022.874031>. PMID: 35602724; PMCID: PMC9114475.

8.Zhai BY, Li DP, Jia JC, Liu YX, Sun WQ, Wang YH. Peer victimization and problematic internet use in adolescents: The mediating role of deviant peer affiliation and the moderating role of family functioning. Addict Behav. 2019; 96:43-49. <https://doi.org/10.1016/j.addbeh.2019.04.016>. PMID: 31035077.

9.Koronczai B, Kökönyei G, Griffiths MD, Demetrovics Z. The relationship between personality traits, psychopathological symptoms, and problematic internet use: A complex mediation model. J Med Internet Res. 2019;21(4):e11837. <https://prod7.jmir.org/2019/4/e11837>. PMID: 31025955.

35.Zhang RP, Bai BY, Jiang S, Yang S, Zhou Q. Parenting styles and internet addiction in Chinese adolescents: Conscientiousness as a mediator and teacher support as a moderator. Comput Hum Behav.2019; *101*, 144-150. <https://doi.org/10.1016/j.chb.2019.07.019>.

45.Stavrulaki E, Li M, Gupta J. Perceived parenting styles, academic achievement, and life satisfaction of college students: The mediating role of motivation orientation. Eur J Psychol Educ. 2021; 36, 693–717. <https://doi.org/10.1007/s10212-020-00493-2>.

54.Baloğlu M, Şahin R, Arpaci I. A review of recent research in problematic internet use: gender and cultural differences. Curr Opin Psychol. 2020; 36:124-129. <https://doi.org/10.1016/j.copsyc.2020.05.008>. PMID: 32629412.

55.Lin X, Gu JY, Guo WJ, Meng YJ, Wang HY, Li XJ, et.al. The gender-sensitive social risk factors for Internet Addiction in college undergraduate students. Psychiatry Investig. 2021; 18(7): 636-644. <https://doi.org/10.30773/pi.2020.0277>. PMID: 34340274.

59.Joshanloo M, Jovanović V. The relationship between gender and life satisfaction: analysis across demographic groups and global regions. Arch Womens Ment Health. 2020;23(3):331-338. <https://doi.org/10.1007/s00737-019-00998-w>. PMID: 31482245.

60.Henkens JHD, Kalmijn M, de Valk HAG. Life satisfaction development in the transition to adulthood: Differences by gender and immigrant background. J Youth Adolesc. 2022; 51(2): 305-319. <https://doi.org/10.1007/s10964-021-01560-7>. PMID: 35024977.

**Comments 4: Lines 41-42, as far as I know, there is not a few literature related to parenting style, life satisfaction and Internet addiction. Please cite the appropriate literature of recent years to support this statement.**

**Response to comments 4:**

As suggested by the reviewer, we have adjusted the **Introduction** by deleting lines 41-42 and moving them to lines 60-64, 64-66 (revised manuscript with track changes; see lines 47-50, 51-54 in manuscript). Moreover, we have added some new references about to support this statement.

References:

7.Zhao Y, Zhang K, Griffiths MD. Serial mediation roles of Alexithymia and loneliness in the association between family function and internet addiction among Chinese college students. Front Psychol. 2022; 13: 874031. <https://doi.org/10.3389/fpsyg.2022.874031>. PMID: 35602724; PMCID: PMC9114475.

8.Zhai BY, Li DP, Jia JC, Liu YX, Sun WQ, Wang YH. Peer victimization and problematic internet use in adolescents: The mediating role of deviant peer affiliation and the moderating role of family functioning. Addict Behav. 2019; 96:43-49. <https://doi.org/10.1016/j.addbeh.2019.04.016>. PMID: 31035077.

9.Koronczai B, Kökönyei G, Griffiths MD, Demetrovics Z. The relationship between personality traits, psychopathological symptoms, and problematic internet use: A complex mediation model. J Med Internet Res. 2019;21(4):e11837. <https://prod7.jmir.org/2019/4/e11837>. PMID: 31025955.

10.Zhang, R., Bai, B., Jiang, S., Yang, S., & Zhou, Q. Parenting styles and internet addiction in Chinese adolescents: Conscientiousness as a mediator and teacher support as a moderator. Comput Hum Behav. 2019; 101, 144-150. <https://doi.org/10.1016/j.chb.2019.07.019>.

11.Guo YN, Chen HY, You X, Liu XF, Sun XQ, Jin YC. Relationship between parenting style and internet addiction: Interpersonal relationship problem as a mediator and gender as a moderator. Heliyon. 2024; 10(2):e 23973. <https://doi.org/10.1016/j.heliyon.2024.e23973>. PMID: 38293521.

12.Lukavská K, Vacek J, Gabrhelík R. The effects of parental control and warmth on problematic internet use in adolescents: A prospective cohort study. J Behav Addict. 2020;9(3):664-675. <https://doi.org/10.1556/2006.2020.00068>. PMID: 32976113

14.Xiong A, Liao S, Luo B, Luo S, Tong Y, Li Z. Associations between problematic internet use, life satisfaction, and deliberate self-harm among Chinese adolescents: A multi-centered longitudinal study. Addict Behav. 2023; 147:107808. <https://doi.org/10.1016/j.addbeh.2023.107808>. PMID: 37536220.

**Comments 5: The logic of the Introduction needs to be adjusted and supplemented, and several subheadings need to be clarified to clearly state the relationships between the various variables, the theoretical foundations, and the hypotheses of the study.**

**Response to comments 5:**

Thank you for your suggestions on improving the manuscript. We have reorganized the logic based on the original manuscript, carefully adjusted and modified the structure and logic of the **Introduction** of the manuscript. In the **Introduction**, we have introduced the research objective, and then elaborate on the main theoretical model used in this study. Then, we have explored the current situation of **parenting styles and internet addition**, **the mediating role of life satisfaction**, and **gender differences**. Finally, we have integrated the previous discussion and propose the hypothesis of this study in **The present study** part. In **The present study**, we proposed two hypotheses for this study: Life satisfaction has a mediating role in the influence relationship of parenting styles on internet addiction among Chinese college students (H1). The mechanism of mediation in different gender groups should be different. (H2).

**Comments 6: Lines 97 and 99, “Previous studies........ , multiple studies......”, but to cite only one study as support is very loose. This kind of situation occurs in many places in the manuscript, and this problem is very bad and needs to be paid attention to by the author.**

**Response to comments 6:**

We are sorry for our careless mistakes. We have carefully revised the relevant parts according to the manuscript, some adding more references to support, and some modifying the words.

Add references:

See lines: 63, 70, 75, 114, 131, 145, 160, 197, 209, 210, 464, 547, 569. (revised manuscript with track changes)

See lines: 50, 57, 61, 91, 103, 113, 123, 149, 159, 160, 371, 377, 458, 474. (manuscript)

Revised:

Lines 109, 497. (revised manuscript with track changes)

Lines 89, 410. (manuscript)

10.Zhang, R., Bai, B., Jiang, S., Yang, S., & Zhou, Q. Parenting styles and internet addiction in Chinese adolescents: Conscientiousness as a mediator and teacher support as a moderator. Comput. Hum. Behav. 2019; 101, 144-150. <https://doi.org/10.1016/j.chb.2019.07.019>

11.Guo YN, Chen HY, You X, Liu XF, Sun XQ, Jin YC. Relationship between parenting style and internet addiction: Interpersonal relationship problem as a mediator and gender as a moderator. Heliyon. 2024; 10(2):e 23973. <https://doi.org/10.1016/j.heliyon.2024.e23973>. PMID: 38293521; PMCID: PMC10826173.

12.Lukavská K, Vacek J, Gabrhelík R. The effects of parental control and warmth on problematic internet use in adolescents: A prospective cohort study. J Behav Addict. 2020;9(3):664-675. <https://doi.org/10.1556/2006.2020.00068>. PMID: 32976113.

35.Zhang RP, Bai BY, Jiang S, Yang S, Zhou Q. Parenting styles and internet addiction in Chinese adolescents: Conscientiousness as a mediator and teacher support as a moderator. Comput. Hum Behav.2019; 101, 144-150. <https://doi.org/10.1016/j.chb.2019.07.019>.

36.Niu X, Li JY, King DL, Rost DH, Wang HZ, Wang JL. The relationship between parenting styles and adolescent problematic Internet use: A three-level meta-analysis. J Behav Addict. 2023;12(3):652–69. <https://doi.org/10.1556/2006.2023.00043>. PMID: 37725505; PMCID: PMC10562819.

40.Smith CA, Konik J. Who is satisfied with life? Personality, cognitive flexibility, and life satisfaction. Curr Psychol. 2022; 41, 9019–9026. <https://doi.org/10.1007/s12144-021-01359-6>.

41.Mahamid FA, Berte DZ, Bdier D. Problematic internet use and its association with sleep disturbance and life satisfaction among Palestinians during the COVID-19 pandemic. Curr Psychol. 2022; 41(11): 8167-8174. <https://doi.org/10.1007/s12144-021-02124-5>. PMID: 34334988; PMCID: PMC8310401.

43.Sun Y, Wang MP, Chan CS, Lo DLO, Wan ANT, Lam TH, et.al. Promoting positive parenting and mental wellbeing in Hong Kong Chinese parents: A pilot cluster randomised controlled trial. PLoS One. 2022;17(7):e0270064. <https://doi.org/10.1371/journal.pone.0270064>. PMID: 35857769; PMCID: PMC9299310.

44.Di Giunta L, Lunetti C, Gliozzo G, Rothenberg WA, Lansford JE, Eisenberg N, et.al. Negative parenting, adolescents' emotion regulation, self-efficacy in emotion regulation, and psychological adjustment. Int J Environ Res Public Health. 2022; 19(4): 2251. <https://doi.org/10.3390/ijerph19042251>. PMID: 35206436; PMCID: PMC8871997.

52.Hà TA, Tran MAQ, Lin CY, Nguyen QL. Facebook addiction and high school students' sleep quality: The serial mediation of procrastination and life satisfaction and the moderation of self-compassion. J Genet Psychol. 2023; 184(6): 415-429. <https://doi.org/10.1080/00221325.2023.2219717>. PMID: 37350555.

55.Lin X, Gu JY, Guo WJ, Meng YJ, Wang HY, Li XJ, et.al. The gender-sensitive social risk factors for Internet addiction in college undergraduate students. Psychiatry Investig. 2021; 18(7): 636-644. <https://doi.org/10.30773/pi.2020.0277> PMID: 34340274.

59.Joshanloo M, Jovanović V. The relationship between gender and life satisfaction: analysis across demographic groups and global regions. Arch Womens Ment Health. 2020;23(3):331-338. <https://doi.org/10.1007/s00737-019-00998-w>. PMID: 31482245.

**Comments 7: Please clarify the research questions and the study hypotheses.**

**Response to comments 7:**

We have carefully revised this section and added a new part, **The present study**, to clarify the hypotheses of this study. In **The present study**, we proposed two hypotheses for this study: Life satisfaction has a mediating role in the influence relationship of parenting styles on internet addiction among Chinese college students (H1). The mechanism of mediation in different gender groups should be different. (H2).

Revised content:

See lines:243-258. (revised manuscript with track changes)

See lines:187-198. (manuscript)

**Comments 8: If the hypothesis model is to explore the dimensions, the literature review on the relationship between the dimensions and the main variables needs to be supplemented in the Introduction.**

**Response to comments 8:**

Thank you for pointing out the shortcomings of our literature review. We have carefully revised and added the literature review on dimensions and main research variables. The literature review on the relationship between dimensions of family parenting styles and internet addiction is in Lines 131-144 (revised manuscript with track changes); The literature review on the dimensions and life satisfaction is in Lines 174-176, 181-183 (revised manuscript with track changes). And we added more references on the relationship between the dimensions and the main variables.

Revised content:

See lines:103-112, 134-136, 138-141. (manuscript)

**Comments 9: The data for this study were collected from September to October 2021, and it is currently 2024, so the timeliness of the data is worth considering. In addition, what is the serial number of ethical approval?**

**Response to comments 9:**

Firstly, we have added the serial number of ethical approval to the manuscript, which is 2021NO.07 for this study.

Secondly, the reviewer has doubts about the timeliness of the data in this study. This study is a comprehensive series of studies on internet addiction, and so far, research on internet addiction is still ongoing. This study is an in-depth exploration and comprehensive analysis of preliminary data.

Revised content:

See line:278 (revised manuscript with track changes)

See line:211 (manuscript)

**Comments 10: The data is collected through the psychological system. Is there a valid link to this system?**

**Response to comments 10:**

We apologize for the wording error. This study used a paper questionnaire, which was distributed and collected offline. We have added the process of questionnaire collection for this study to the **Procedures**. Meanwhile, we have added some original paper questionnaires on the last page of this letter.

Revised content:

See lines:322-326 (revised manuscript with track changes)

See lines:247-252 (manuscript)

**Comments11: Please provide sample items for each scale.**

**Response to comments 11:**

Thank you for your suggestion. We have attached sample items for Chinese version of each scale below, and have also included some paper questionnaires at the end of the letter.

| 这是一份有关网络成瘾的调查问卷，请您按照实际情况作答，答案没有对错之分 | | | 非常符合 | 符合 | 不符合 | | 极不符合 | |
| --- | --- | --- | --- | --- | --- | --- | --- | --- |
| 1.曾不止一次有人告诉我，我花太多时间在网络上 | | |  |  |  | |  | |
| 2.如果有一段时间不上网，就会觉得心里不舒服 | | |  |  |  | |  | |
| 3.我发现自己上网的时间越来越长 | | |  |  |  | |  | |
| 4.断线或接不上网络时，我觉得自己坐立不安 | | |  |  |  | |  | |
| 5.再累，上网时都觉得自己很有精神 | | |  |  |  | |  | |
| 6.我每次都只想上网待一下子，但常常一待就很久不想下来 | | |  |  |  | |  | |
| 7.虽然上网对我日常与同学，家人的人际关系造成负面影响，我仍未减少上网 | | |  |  |  | |  | |
| 8.我曾不止一次因为上网的关系一天睡眠时间不到四小时 | | |  |  |  | |  | |
| 以下是有关父母教养方式的部分，请参考您的实际情况作答，答案没有对错之分 | | | 从不 | 偶尔 | 经常 | | 总是 | |
| 1.我觉得父母干涉我所做的每一件事 | | | 父亲 |  |  | |  | |
|  |  |  | 母亲 |  |  | |  | |
| 2.我能通过父母的言读、表情感受他(她)很喜欢我 | | | 父亲 |  |  | |  | |
|  |  |  | 母亲 |  |  | |  | |
| 3.与我的兄弟姐妹比，父母更宠爱我 | | | 父亲 |  |  | |  | |
|  |  |  | 母亲 |  |  | |  | |
| 4.我能感到父母对我的喜爱 | | | 父亲 |  |  | |  | |
|  |  |  | 母亲 |  |  | |  | |
| 这是一份有关生活满意度的调查问卷，请您按实际情况作答。 | 非常不同意 | 不同意 | 有点不同意 | 没有  意见 | 有点同意 | 同意 | | 非常同意 |
| 1.我的生活大致符合我的理想 |  |  |  |  |  |  | |  |
| 2.我的生活状况非常圆满 |  |  |  |  |  |  | |  |
| 3.我满意自己的生活 |  |  |  |  |  |  | |  |
| 4.直到现在为止，我都能够得到  在生活上希望拥有的重要东西 |  |  |  |  |  |  | |  |
| 5.如果可以重新选择生活，我几乎不会选择改变现状 |  |  |  |  |  |  | |  |

**Comments 12: Please specify the process of questionnaire collection and how to identify invalid questionnaires?**

**Response to comments 12:**

Thank you for your valuable feedback and questions regarding the collection of this research questionnaire. This study contacted various professional psychological commissioners through the college psychological organization and collected paper questionnaires offline. Two psychology graduate students and one psychological commissioner from each major jointly distributed and collected questionnaires. The two psychology graduate students read out guidance before distributing the questionnaires, and after distribution, they were collected uniformly.

Secondly, we have added the invalid questionnaire criteria the process of questionnaire collection for this study to the **Procedures**.

In this study, if the number of questions filled in the questionnaire is less than one-third, it will be considered invalid.

Revised content:

See lines:322-326, 329-330. (revised manuscript with track changes)

See lines:247-252, 253-254. (manuscript)

**Comments13: Authors only provide reliability, whether the validity meets the criteria, please add.**

**Response to comments 13:**

Thank you for your suggestions on improving the manuscript. We have added the content of validity analysis for this study. This study analyzed the structural validity of each scale and added correlations based on the MI coefficient. Finally, it was shown that all pathways of each scale were statistically significant. Based on the statistical indicators proposed by Fornell et al., Doll WJ et al., and Hooper D et al., the final fit indices of the structural validity model in this study were acceptable. In this study, the final structural validity fit indices of each scale were as follow: maternal parenting style(*χ^2^/df*= 3.367, RMSEA=0.049, GFI=0.828, SRMR=0.090, TLI=0.843), paternal parenting style (*χ^2^/df* =3.449, RMSEA=0.050, GFI=0.820, SRMR=0.090, TLI=0.833), internet addiction (*χ^2^/df*=4.677, RMSEA =0.062, GFI=0.891, SRMR=0.046, TLI=0.879) and life satisfaction(*χ^2^/df*=4.439, RMSEA=0.032, GFI=0.992, SRMR=0.014, TLI=0.989).

The final structural model diagram is attached below (Fig 1-4)

The results have been added to the **Measures** of the manuscript; The reference indicators have been added to the **Data Analysis**.

Revised content:

CFA results:

See lines:296-298, 305-308, 316-319 (revised manuscript with track changes)

See lines:226-229, 233-236, 243-245 (manuscript)

Reference indicators:

See lines:339-343(revised manuscript with track changes)

See lines:263-266(manuscript)

Fig 1 maternal parenting style

Fig 2 paternal parenting style

Fig 3 paternal parenting style

Fig 4 life satisfaction

**Comments 14: Please provide valid references for the standard values of all indicators in the Results section, such as Common method deviation test (180 lines), etc.**

**Response to comments 14:**

Thank you to the reviewer for providing valuable feedback. According to this suggestion, we have added references to the standards for statistical analysis of the manuscript. We have added reliability indicators, structural validity indicators, common method bias indicators, and references. All references for the standard values have been added to the **Data Analysis**.

Revised content:

See lines: 337-343, 346-348 (revised manuscript with track changes)

See lines: 262-266, 270-271 (manuscript)

Add references:

64.Churchill GA. A paradigm for developing better measures of marketing constructs. J Marketing Res. 1979; 16(1), 64–73. [https://doi.org/10.2307/3150876](https://psycnet.apa.org/doi/10.2307/3150876).

65.Fornell C, Larcker DF. Structural equation models with unobservable variables and measurement Error: Algebra and statistics. J Marketing Res. 1981; 18(3), 382-388. <https://doi.org/10.1177/002224378101800313>.

66.Doll WJ, Xia W, Torkzadeh G. A confirmatory factor analysis of the end-user computing satisfaction instrument. Mis Quart; 18(4): 453–461. <https://doi.org/10.2307/249524>.

67.Hooper D, Coughlan J, Mullen MR. Structural equation modeling: Guidelines for determining model fit. Electron J on Bus Res Methods. 2008; 6(1):53-60.<https://doi.org/10.21427/D7CF7R>.

68.Hau KT, Wen ZL, Cheng ZJ. Structural Equation Model and Its Application. Beijing: Educational Science Publishing House; 2004.

69.Podsakoff PM, MacKenzie SB, Lee J-Y, Podsakoff NP. Common method biases in behavioral research: a critical review of the literature and recommended remedies. J Appl Psychol. 2003; 88 (5):879. <https://doi.org/10.1037/0021-9010.88.5.879>. PMID: 14516251.

**Comments 15: Please add practical suggestions.**

**Response to comments 15:**

Thank you for your suggestions on improving the manuscript. We tried our best to improve the manuscript and provided more specific suggestions. We have made modifications to the suggestions in the **Discussion**.

This study might be helpful for parents to rethink about their parenting styles, such as improving or adjusting their parenting behavior to reduce the risk of adolescent internet addiction. Parents should care about and support adolescents, develop positive interactions with them, praise them. Meanwhile, the influence of parenting styles on college students' internet addiction is also influenced by the factor of life satisfaction. This suggests that in the future, prevention and intervention for college students' internet addiction should help college students improve their own life satisfaction, for example, helping to improve emotional regulation, relax, and increase effective interpersonal communication. Moreover, when focusing on the work of both males and females, attention should be paid to the impact of different parenting dimensions on individuals’ internet addiction. In conclusion, this study suggests that in the future, more attention should be paid to the role of family factors in solving the internet addiction among college students. Starting from the cooperation between families and schools, the prevalence of internet addiction among college students may be reduced.

Revised content:

See lines: 574-583,587-592 (revised manuscript with track changes)

See lines: 477-485,492-496 (manuscript)

**Comments 16: Please specify exactly how future research should proceed in the Limitations.**

**Response to comments 16:**

Thank you for your suggestion about **Limitations**. We have carefully supplemented the content that could be improved for future research in **Limitations**. In **Limitations**, regarding the sampling issue, we think that in the future, we can expand the source of samples and collect samples from multiple universities and wider regions. Secondly, it is difficult to draw causal conclusions through cross-sectional studies in this study. In the future, a longitudinal tracking study can be used to improve the research.

Revised content:

See lines: 599-600,602 (revised manuscript with track changes)

See lines: 500-501,503 (manuscript)

**Comments 17: The findings, contributions and implications of this study need to be summarized in the concluding section.**

**Response to comments 17:**

Thank you for your suggestions on improving the manuscript. We have carefully revised the **Conclusion**, integrating and expressing the objective of this study, as well as the results and recommendations obtained from the study.

The **Conclusion** is as follows. This study mainly explored the potential mechanisms of internet addiction, the impact of family parenting styles on college students’ internet addiction, the mediating role of life satisfaction, and the role of gender in it. Parenting styles, particularly that of the father, have a major effect on students’ internet addiction, which is partially mediated by life satisfaction. For students of different genders, life satisfaction has different mediating effects, and the influence of parenting styles also present different forms. These findings indicate that paying attention to the role of the family, especially the parenting style of fathers, is crucial for preventing internet addiction in the future. This study may help parents rethink their parenting styles, such as improving or adjusting their parenting behavior based on gender, to reduce the risk of adolescent internet addiction.

Revised content:

See lines: 609-611,615-619 (revised manuscript with track changes)

See lines: 510-512,516-520 (manuscript)

Finally, regarding the reviewer's suggestion on the reference doi, we have added the relevant literature's doi.

See lines: 730,734,832,853 (revised manuscript with track changes)

See lines: 625,629,723,744 (manuscript)

References:

29.Bae SM. The relationships between perceived parenting style, learning motivation, friendship satisfaction, and the addictive use of smartphones with elementary school students of South Korea: Using multivariate latent growth modeling. Sch Psychol Int. 2015; 36(5), 513–531. <https://doi.org/10.1177/0143034315604017>.

30.Hsieh Y, Shen AC, Wei H, Feng J, Huang SC, Hwa H. Internet addiction: A closer look at multidimensional parenting practices and child mental health. Cyberpsychol Behav Soc Netw. 2018; 21, 768–773. <https://doi.org/10.1089/cyber.2018.0435>.

56.Toker S, Baturay MH. Antecedents and consequences of game addiction. Comput Hum Behav. 2016; 55(Part B), 668–679. <https://doi.org/10.1016/j.chb.2015.10.002>

62.Diener E, Emmons RA, Larsen RJ, Griffin S. The satisfaction with life scale. J Pers Assess. 1985; 49(1), 71–75. <https://doi.org/10.1207/s15327752jpa4901_13>.

**#Reviewer 2:**

**Comments 1: No strong arguments for mediation are presented.**

**Response to comments 1:**

Thank you for pointing out the research shortcomings. We think this is an excellent suggestion. In the **Introduction**, we did not express the relationship between the main variables in depth and the logic was not clear. Therefore, we readjusted and reorganized the relationship between the main variables. We have adjusted and revised the research review content in **The mediating role of life satisfaction** (lines 159-204; revised manuscript with track changes) of this study. In this part, we respectively elaborated the relationship between family parenting styles and life satisfaction, and the relationship between Internet addiction and life satisfaction. Then, based on the theoretical model of this study: the cognitive-behavioral model of pathological internet use, and previous studies on life satisfaction as a mediating factor, we proposed the mediation model hypothesis of this study.

Revised content:

See lines: 159-165, 172-180, 196-204 (revised manuscript with track changes)

See lines: 123-129, 130-140, 148-156 (manuscript)

**Comments 2: Moreover, hypotheses are missing.**

**Response to comments 2:**

We have carefully revised this part and added a new section, **The present study**, to clarify the hypotheses of this study.

In **The present study**, we proposed two hypotheses for this study: Life satisfaction has a mediating role in the influence relationship of parenting styles on internet addiction among Chinese college students (H1). The mechanism of mediation in different gender groups should be different. (H2).

Revised content:

See lines:244-258. (revised manuscript with track changes)

See lines:186-198. (manuscript)

**Comments 3: Each parental style may be defined and linked with the other main study variables. In the introduction, there are several information about authoritarian, tolerant, positive parental behaviors. However, the instrument measure emotional warmth, punitiveness, over-involvement, preference, rejection, and overprotection, but not reference to these styles and the relation with life satisfaction and internet addiction is made in the introduction.**

**Response to comments 3:**

Thank you for pointing out the shortcomings of the manuscript. Firstly, after reviewing the literature, we have added a classification of the parenting styles investigated in this study in the **Introduction**, and also explained some parenting styles mentioned in the study that are different from the research styles. We have divided parenting styles into positive and negative according to the previous study, with authoritative style and emotional warmth being classified as positive parenting, and authoritarian, tolerant, overprotection, over involvement, rejection being classified as negative parenting. This section has been added to the **Introduction**.

Revised content:

See lines:93-96. (revised manuscript with track changes)

See lines:79-82. (manuscript)

**Comments 4: Moreover, the differential role of mother and father in predicting life satisfaction and internet addiction should also be discussed in the Introduction.**

**Response to comments 4:**

We appreciate your insightful feedback regarding the need for adding content. We have carefully revised and added the review of fathers and mothers predicting life satisfaction and internet addiction. After consulting literature, it can be seen that the different parenting styles of fathers and mothers have different effects on internet addiction and life satisfaction. Therefore, we have added new supplements and explanations to this section in the manuscript.

Revised content:

Parenting styles affect internet addiction:

See lines:144-150. (revised manuscript with track changes)

See lines:112-118. (manuscript)

Parenting styles affect life satisfaction:

See lines:187-195. (revised manuscript with track changes)

See lines:141-147. (manuscript)

**Comments 5: For what reasons some questionnaires are considered invalid?**

**Response to comments 5:**

In this study, if the number of questions filled in the questionnaire is less than one-third, it will be considered invalid.

We have added the invalid questionnaire criteria for this study to the **Procedures**.

Revised content:

See lines:329-330 (revised manuscript with track changes)

See lines:253-254 (manuscript)

**Comments 6: For the instruments, Alpha Cronbach need to be reported for each scale.**

**Response to comments 6:**

We have calculated the Cronbach’ α for each dimension of EMBU and each dimension of CIAS-R. The Cronbach’s α for each dimension of EMBU are 0.920, 0.900, 0.714, 0.775, 0.782, 0.629, 0.934, 0.809, 0.839, 0.884, 0.770, respectively. The Cronbach’s α for each dimension of CIAS-R are 0.763, 0.761, 0.708, 0.834, 0.757, respectively. And we have added these to **Measures** in the manuscript.

Revised content:

See lines:291-292, 314-315(revised manuscript with track changes)

See lines:223-224, 242(manuscript)

**Comments 7: Moreover, the results of factorial analysis for the multidimensional scales should be presented in the Method section.**

**Response to comments 7:**

Thank you for your suggestions on improving the manuscript. We have added the content of validity analysis for this study. This study analyzed the structural validity of each scale and added correlations based on the MI coefficient. Finally, it was shown that all pathways of each scale were statistically significant. Based on the statistical indicators proposed by Fornell et al., Doll WJ et al., and Hooper D et al., the final fit indices of the structural validity model in this study were acceptable. In this study, the final structural validity fit indices of each scale were acceptable: maternal parenting style (*χ^2^/df*=3.367, RMSEA=0.049, GFI=0.828, SRMR=0.090, TLI=0.843), paternal parenting style (*χ^2^/df* =3.449, RMSEA=0.050, GFI=0.820, SRMR=0.090, TLI=0.833) and internet addiction (*χ^2^/df*=4.677, RMSEA=0.062, GFI=0.891, SRMR=0.046, TLI=0.879).

The final structural model diagram is attached below (Fig 1-3)

The results have been added to the **Measures** of the manuscript; The reference indicators have been added to the **Data Analysis**.

Revised content:

CFA results:

See lines:296-300, 316-319 (revised manuscript with track changes)

See lines:226-229, 243-245 (manuscript)

Reference indicators:

See lines:339-343(revised manuscript with track changes)

See lines:263-266(manuscript)

Fig 1 maternal parenting style

Fig 2 paternal parenting style

Fig 3 paternal parenting style internet addiction

**Comments 8: More clarity is needed in the presentation of the Results. This section can be organized according to the study objectives and hypotheses.**

**Response to comments 8:**

We sincerely thank the reviewer for careful reading. As suggested by the reviewer, we have carefully revised the analysis of the mediation model in the results section based on the revised research hypothesis. Based on hypothesis 1, we have highlighted in the overall mediation analysis that life satisfaction mediates the impact of two parenting styles on internet addiction. According to hypothesis 2, the differences in the influence mechanism of parenting styles among different groups in the analysis of male and female groups were expressed.

Revised content:

see lines 392-397, 419-426 (revised manuscript with track changes)

see lines 313-319, 338-346 (manuscript)

We tried our best to improve the manuscript. We appreciate for Editor’s/Reviewers’ warm work, and hope the correction will meet with approval. Once again, thank you very much for your comments and suggestions.

**Paper questionnaires:**
