# Supplementary material for: Effect of paternal-maternal parenting styles on college students’ internet addiction of different genders: The mediating role of life satisfaction
Source: PLoS One. 2024 May 14;19(5):e0303554. doi: 10.1371/journal.pone.0303554 (PMC11093304; doi:10.1371/journal.pone.0303554)
Supplement: Attachment — Submitted filename: Response to Reviewers.docx [file pone.0303554.s005.docx]

Dear Editor and Reviewers,

We appreciate the thoughtful comments and constructive feedback provided by the Editor and Reviewers. Those comments and feedback are all valuable and very helpful for revising and improving our manuscript. According to the comments and feedback, we have made extensive modifications to the manuscript. The reviewers’ comments are laid out below in **bold font** and specific concerns have been numbered. And changes/additions to the manuscript are identified using the “Track Changes” option in Microsoft Word.

**# Editor:**

**Comments 1. in the results section, please add the coefficients before the *p* value for significance or remove the *p*. It is unusual to find only *p* values.**

**Response to Comments 1:**

Firstly, thank you for taking the time to review our manuscript and your reminder. We have carefully read and responded to every suggestion and evaluation from reviewers.

For the first suggestion, as the reference values are already listed in the table. Therefore, we have carefully considered and removed the *p*-value from the **Result** of the manuscript.

Revised content:

see lines 285-290, 303-311 (revised manuscript with track changes)

see lines 278-283, 296-303 (manuscript)

**Comments 2. Reply to reviewer feedback.**

**Response to Comments 2:**

**In response to the two suggestions from #Reviewer 1**

**Comments 1. First, “This study is a comprehensive series of studies on internet addiction, and so far, research on internet addiction is still ongoing. ” This reason does not explain the timeliness of the data, and cross-sectional studies should also consider the timeliness of the data.**

**Response to comments 1:**

We gladly accept and will try our best to avoid the issues of data timeliness. In future research, we will pay attention to the timeliness of data in cross-sectional studies to avoid such issues from happening again. With new data, we will organize and analyze the data in a timely manner.

**Comments 2. Second, the authors should have taken a more scientific approach to identifying invalid questionnaires. Please study further.**

**Response to comments 2:**

Thank you to the reviewer for letting us discover the shortcomings in data processing. In the future, we will refer to previous research and most of the methods used by scholars to handle and identify invalid questionnaires more cautiously and scientifically.

**In response to the suggestions from #Reviewer 2**

We have carefully made revisions to the manuscript raised by Reviewer 2, regarding the details of the manuscript.

**Comments 1:** I recommend to remove the heading Theory in the Introduction.

**Response to comments 1:**

We are very grateful for the details the reviewer has raised. We have removed the heading **Theory** in the **Introduction** and combined it with the rest to make the logic of the manuscript more coherent.

Revised content:

See lines: 72-80 (revised manuscript with track changes)

See lines: 65-73 (manuscript)

**Comments 2:** Avoid causal terms (e.g., the word influence in hypotheses).

**Response to comments 2:**

We have carefully reviewed the manuscript and revised the causal terms related to the viewpoints proposed in this study and replaced it with words such as association, relationship, and correlation.

Revised content:

See lines: 23, 26-27, 29, 112, 143, 190, 202, 324, 347, 351, 396, 401, 417, 426, 435, 437, 463, 468, 486(revised manuscript with track changes)

See lines: 23, 26, 29, 106, 137, 184, 195, 316, 339, 343, 388, 393, 408, 416, 425, 427, 453, 458, 476(manuscript)

**Comments 3:** The hypotheses could be presented at a specific level, by mentioning the specific mediation expected.

**Response to comments 3:**

We have made specific statements about the assumptions proposed in the manuscript. We have refined the relationship between different types of parenting styles and internet addiction, assuming that the emotional warmth of parenting styles may be positively correlated with internet addiction, while parenting styles are all as overprotection, involvement, punishment, and preference are positively correlated with internet addiction.

Revised content:

See lines: 199-203 (revised manuscript with track changes)

See lines: 192-196 (manuscript)

**Comments 4:** In the results section, please add the coefficients before the *p* value for significance or remove the *p*. It is unusual to find only *p* values.

**Response to comments 4:**

After careful consideration, regarding the *p*-value in the manuscript, as the reference values have been presented in the table. Therefore, we have carefully considered and removed the *p*-value from the **Result** of the manuscript.

Revised content:

See lines: 285-290, 303-311 (revised manuscript with track changes)

See lines: 278-283, 296-303 (manuscript)

**#Reviewer 1:**

Dear author,

Many thanks for your efforts in revising the manuscript. The revised manuscript is greatly improved in all respects. My opinion is that the manuscript is acceptable. But there are still two problems that the authors need to be aware of.

**Response to #Reviewer 1:**

Thank you for taking the time to provide valuable suggestions for improving the manuscript. Your suggestions have greatly helped to improve the research level and ideas of the researchers in this study. Moreover, we are glad to hear your affirmation of the manuscript. We will keep in mind your two remaining questions about the manuscript and consciously avoid such problems from happening again in future research.

**Comments 1. First, “This study is a comprehensive series of studies on internet addiction, and so far, research on internet addiction is still ongoing. ” This reason does not explain the timeliness of the data, and cross-sectional studies should also consider the timeliness of the data.**

**Response to Comments 1:**

Thank you for your suggestions and feedback on data timeliness. In future research, we will pay attention to the timeliness of data in cross-sectional studies to avoid such issues from happening again. With new data, we will organize and analyze the data in a timely manner.

**Comments 2. Second, the authors should have taken a more scientific approach to identifying invalid questionnaires. Please study further.**

**Response to Comments 2:**

Thank you for letting us discover the shortcomings in data processing. In the future, we will refer to previous research and most of the methods used by scholars to handle and identify invalid questionnaires more cautiously and scientifically.

**#Reviewer 2:**

The authors addressed all my concern and the manuscript is significantly improved. I have only some small remarks.

**Comments 1:** I recommend to remove the heading Theory in the Introduction.

**Response to comments 1:**

Thank you for your affirmation of our revised manuscript. We are very grateful for the details you have raised, and these suggestions can help improve the manuscript even better. For Comments 1, we have removed the heading **Theory** in the **Introduction** and combined it with the rest to make the logic of the manuscript more coherent.

Revised content:

See lines: 72-80 (revised manuscript with track changes)

See lines: 65-73 (manuscript)

**Comments 2:** Avoid causal terms (e.g., the word influence in hypotheses).

**Response to comments 2:**

As suggested by the reviewer, we have carefully reviewed the manuscript and revised the causal terms related to the viewpoints proposed in this study and replaced it with words such as association, relationship, and correlation.

Revised content:

See lines: 23, 26-27, 29, 112, 143, 190, 202, 324, 347, 351, 396, 401, 417, 426, 435, 437, 463, 468, 486(revised manuscript with track changes)

See lines: 23, 26, 29, 106,137, 184, 195, 316, 339, 343, 388, 393, 408, 416, 425, 427, 453, 458, 476(manuscript)

**Comments 3:** The hypotheses could be presented at a specific level, by mentioning the specific mediation expected.

**Response to comments 3:**

We agree with the reviewer’s suggestion and have incorporate the recommended changes into manuscript. We have made specific statements about the assumptions proposed in the manuscript. We have refined the relationship between different types of parenting styles and internet addiction, assuming that the emotional warmth of parenting styles may be positively correlated with internet addiction, while parenting styles are all as overprotection, involvement, punishment, and preference are positively correlated with internet addiction.

Revised content:

See lines: 199-203 (revised manuscript with track changes)

See lines: 192-196 (manuscript)

**Comments 4:** In the results section, please add the coefficients before the *p* value for significance or remove the *p*. It is unusual to find only *p* values.

**Response to comments 4:**

Thank you for your valuable feedback on the details of the manuscript. After careful consideration, regarding the *p*-value in the manuscript, as the reference values have been presented in the table. Therefore, we have carefully considered and removed the *p*-value from the **Result** of the manuscript.

Revised content:

See lines: 285-290, 303-311 (revised manuscript with track changes)

See lines: 278-283, 296-303 (manuscript)

Firstly, all researchers in this study are very grateful for the rigorous review of the article by the Editor and the Reviewers. We sincerely appreciate the time and effort invested by the Reviewers and Editor in evaluating our manuscript. We look forward to any additional feedback or suggestions.
